# Joint effects of group sex-ratio and *Wolbachia* infection on female reproductive success in the terrestrial isopod *Armadillidium vulgare*

**DOI:** 10.1186/s12862-019-1391-6

**Published:** 2019-02-28

**Authors:** Margot Fortin, Joël Meunier, Tiffany Laverré, Catherine Souty-Grosset, Freddie-Jeanne Richard

**Affiliations:** 1 Laboratoire Ecologie et Biologie des Interactions, Equipe “Ecologie, Evolution, Symbiose”, UMR CNRS 7267, Université de Poitiers, Bat B8-B35, 6 rue Michel Brunet, TSA 51106, F-86073 Poitiers, Cedex 9 France; 2 Institut de Recherche sur la Biologie de l’Insecte (IRBI), UMR 7261, CNRS, Université de Tours, Tours, France

**Keywords:** Symbiosis, Mate choice, Multiple mating, Breeding successes

## Abstract

**Background:**

In species that reproduce with sexual reproduction, males and females often have opposite strategies to maximize their own fitness. For instance, males are typically expected to maximize their number of mating events, whereas an excessive number of mating events can be costly for females. Although the risk of sexual harassment by males and resulting costs for females are expected to increase with the proportion of males, it remains unknown whether and how parasitic distorters of a host population’s sex-ratio can shape this effect on the fitness of females. Here, we addressed this question using *Armadillidium vulgare* and its parasite *Wolbachia* that alters the sex-ratio of a population. We set up *Wolbachia-*free and *Wolbachia-*infected females in experimental groups exhibiting 100, 80, 50% or 20% females for 1 year, during which we measured changes in survival, fertility and fecundity.

**Results:**

*Wolbachia* infection shaped the effects of both population sex-ratio and reproductive season on female fecundity. Compared to *Wolbachia*-free females, *Wolbachia*-infected females were less likely to be gravid in populations exhibiting an excess of females and did not exhibit the otherwise negative effect of seasons on this likelihood. Group sex-ratio and *Wolbachia* infection have independent effects on other measured traits. Male-biased populations had females both exhibiting the lowest survival rate after 6 months and producing the smallest number of offspring, independent of *Wolbachia* infection. Conversely, *Wolbachia*-infected females had the lowest likelihood of producing at least one offspring, independent of group sex-ratio. *Wolbachia* infection had no effect on female survival rate.

**Conclusions:**

We demonstrated that male-biased sex-ratio and the presence of *Wolbachia* are costly for females due to sexual harassment by males and bacterial infection, respectively. Interestingly, *Wolbachia* infection triggers another negative effect. This effect does not come from an excess of males and its associated sexual harassment of females but instead from a lack of males and the associated risk for females of remaining unmated. Overall, these findings highlight the importance of social pressures and infection on female fitness and provide insights into our general understanding of the joint and opposite effects of these two parameters in the evolution of reproductive strategies.

**Electronic supplementary material:**

The online version of this article (10.1186/s12862-019-1391-6) contains supplementary material, which is available to authorized users.

## Background

Because males and females typically differ in their investment in reproduction, sexual conflict is predicted to emerge in species with sexual reproduction [1]. In this context, males often improve their fitness by maximizing the number of their sexual partners, whereas females do so by being selective toward the quality of their mate [2, 3]. This sex-specific difference in selective pressure has been suggested to lead to sexual harassment by males, a process during which males constrain females for reproduction and which increases the mating costs for females [3–5]. On the one hand, harassment by males may impact female fitness indirectly, for example by forcing females to spend more time and energy avoiding mating instead of investing in other activities such as foraging [6–8]. On the other hand, harassment by males may have a direct impact on female fitness; for instance, in the bean weevil *Callosobruchus maculatus*, the genitalia of males have been shown to contain many sclerotized spines, damaging genitalia of females during copulation and therefore increasing the mortality of females after multiple mating events [4]. Direct costs have also been reported in the sea lion *Phocarctos hookeri*, where sexual harassment by males increases both female mortality and the associated costs of pup separation [8].

The expression of sexual harassment by males and its resulting costs for females may strongly depend on the proportion of males in a population, as well as on the presence of parasitic distorters, such as the symbiotic bacteria *Wolbachia*, on the host population’s sex-ratio. For example, the importance of male-biased sex-ratio on the sexual harassment of females has been shown in the water strider *Aquarius remiges*. In this species, an artificially male-biased sex-ratio increased the quantity of sexual harassment by males, which in turn modified the mating behavior of females; they invested more time in escape behaviors from males by reducing their foraging time [7]. Conversely, numerous studies have demonstrated the broad impacts of *Wolbachia* – a group of intracellular alpha proteobacteria – on multiple aspects of the biology of their hosts, including their reproductive strategies and outcomes [9]. *Wolbachia* are some of the most prevalent gram-negative bacteria in arthropods [10]. They are vertically transmitted and highly manipulative symbionts [11–14], which trigger multiple effects on the reproduction of their host, such as the emergence of cytoplasmic incompatibilities, induction of parthenogenesis, killing of males and feminization of genetic males [15]. In the terrestrial isopod *Armadillidium vulgare* (Crustacea, Isopoda), *Wolbachia* infected individuals suffer from important fitness costs, such as reduced density and survival of hemocytes [16], lower learning and memory performances, lower copulation investment [17], and reduced preference of infected females by males [18]. In this species, *Wolbachia* is also known to induce the feminization of genetic males by transforming them into phenotypic and functional females [19, 20]. Variation in the prevalence of *Wolbachia* infection across natural populations could thus shape their sex-ratio [15], and *Wolbachia* could therefore have important effects on the expression of sexual harassment by males and on its resulting costs for females.

In this study, we manipulated male-biased sex-ratios and *Wolbachia* infection in four types of experimental groups to test their independent and simultaneous effects on the survival and reproduction of *A. vulgare* females. In this species, females can produce several clutches from only one copulation [21], even if multiple mating frequently occurs [22–24]. Females also have several periods of receptivity throughout their life, as well as exhibit refractory periods just after a first mating and after a first breeding season [24, 25]. At the same time, males can mate with numerous females in a few days [26].

We maintained *Wolbachia-*free or *Wolbachia-*infected females in experimental groups including either 100, 80, 50% or 20% females, and then recorded their survival rate, fertility (gravidity) and fecundity (offspring number) over the following 12 months. If sexual harassment by males is overall costly for females while increasing their mating probability (by providing more mating partners and thus increasing overall sperm availability), we predicted that increased male density should overall negatively affect female survival and increase both their fertility and fecundity. Because *Wolbachia* negatively affects the immune system and survival of its hosts [16], we expected the additional energetic costs of reproductive effort (e.g., male injury, physiological changes due to offspring development) to be reduced in *Wolbachia-*free compared to *Wolbachia-*infected females for survival, fertility and fecundity.

## Results

### Effects of group sex-ratio and *Wolbachia* infection on the survival rate and reproductive success of females

#### Female survival rates

**Fig. 1:**
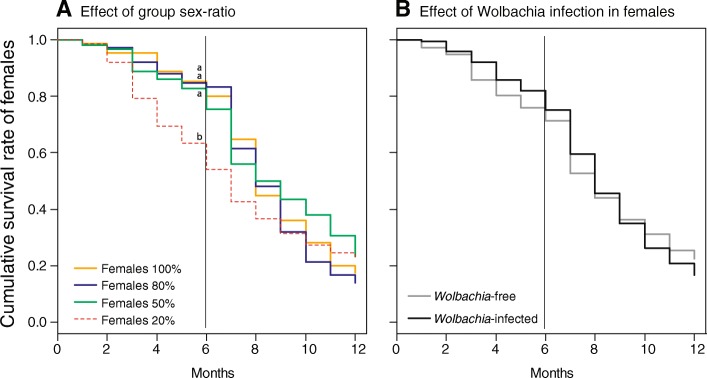
Cumulative survival rates of females over 12 months as a function of (**a**) group sex-ratio and (**b**) *Wolbachia* infection in females. Different letters represent *P* < 0.001 six months after the beginning of the experiment

Over the first 6 months of the experiment, the survival rate of females depended on the group sex-ratio (Fig. 1a; Likelihood Ratio (LR) χ^2^_3_ = 40.74, *P* < 0.0001) but was independent of *Wolbachia* infection (Fig. 1b; LR χ^2^_1_ = 1.76, *P* = 0.1849) and the interaction between group sex-ratio and *Wolbachia* infection (LR χ^2^_3_ = 7.04, *P* = 0.071). In particular, Females _20%_ died overall faster than Females _50%_ (model contrasts; LR χ^2^_1_ = 14.58, *P* = 0.0001), Females _80%_ (LR χ^2^_1_ = 26.54, *P* < 0.0001) and Females _100%_ (LR χ^2^_1_ = 22.26, *P* < 0.0001). The survival rates of Females _50%,_ Females _80%_ and Females _100%_ were similar during this time period (Females _50%_ vs Females _80%:_ LR χ^2^_1_ = 2.70, *P* = 0.100; Females _50%_ vs Females _100%:_ LR χ^2^_1_ = 1.08, *P* = 0.300; Females _80%_ vs Females _100%:_ LR χ^2^_1_ = 0.39, *P* = 0.530). Interestingly, these effects vanished when the overall 12 months of the experiment were considered. In this case, the survival of females became independent of group sex-ratio (LR χ^2^_3_ = 4.05, *P* = 0.2558), *Wolbachia* infection (LR χ^2^_1_ = 0.31, *P* = 0.5755) and the interaction between group sex-ratio and *Wolbachia* infection (LR χ^2^_3_ = 2.67, *P* = 0.4447).

#### Proportion of gravid females

**Fig. 2:**
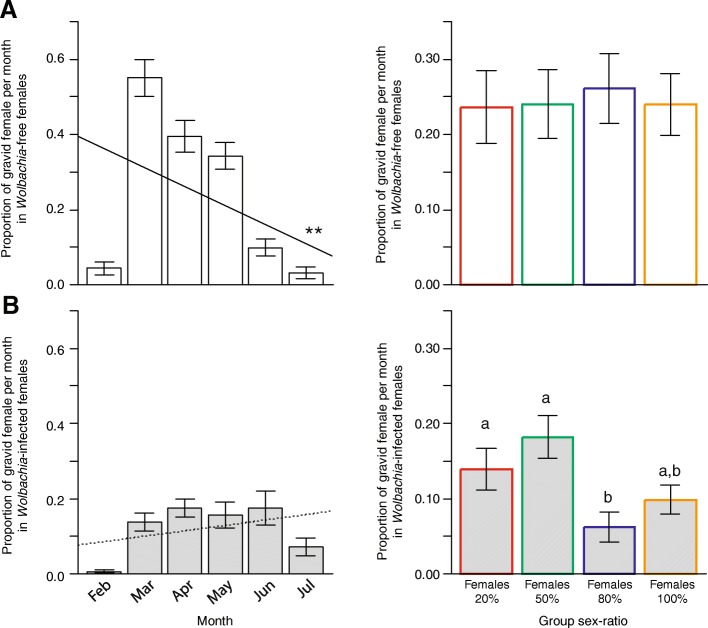
Ratio of gravid females as a function of month (left) and group sex-ratio (right) in (**a**) *Wolbachia*-free and (**b**) *Wolbachia*-infected females. Mean ± SE are presented. Different letters correspond to *P* < 0.05; *** *P* < 0.0001

The proportion of gravid females was shaped by a triple interaction between *Wolbachia* infection, group sex-ratio and month (LR χ^2^_3_ = 8.96, *p* = 0.0297; see full model results in Additional file 1: Table S1). This interaction revealed that in *Wolbachia*-free females, the proportion of gravid females overall decreased over time (Fig. 2a; LR χ^2^_1_ = 8.59, *P* = 0.0034; model estimate ± SE = − 0.042 ± 0.013) but was independent of group sex-ratio (LR χ^2^_3_ = 0.15, *P* = 0.9849) and the interaction between group sex-ratio and month (LR χ^2^_3_ = 4.39, *P* = 0.2227). In contrast, in *Wolbachia*-infected females, the proportion of gravid females did not vary over time (Fig. 2b; LR χ^2^_1_ = 3.53, *P* = 0.0602) but reflected group sex-ratio overall (LR χ^2^_3_ = 13.75, *P* = 0.0033). In particular, the proportion of gravid females was lower in Females _80%_ compared with Females _20%_ (Tukey contrasts, *P* = 0.0299) and Females _50%_ (Tukey contrasts, *P* = 0.0013), whereas all the other pairwise comparisons were nonsignificant (Fig. 2a; all *P* > 0.1450). The proportion of gravid *Wolbachia*-infected females was independent of the interaction between group sex-ratio and month (LR χ^2^_3_ = 7.65, *P* = 0.0539).

#### Production of descendants

**Fig. 3:**
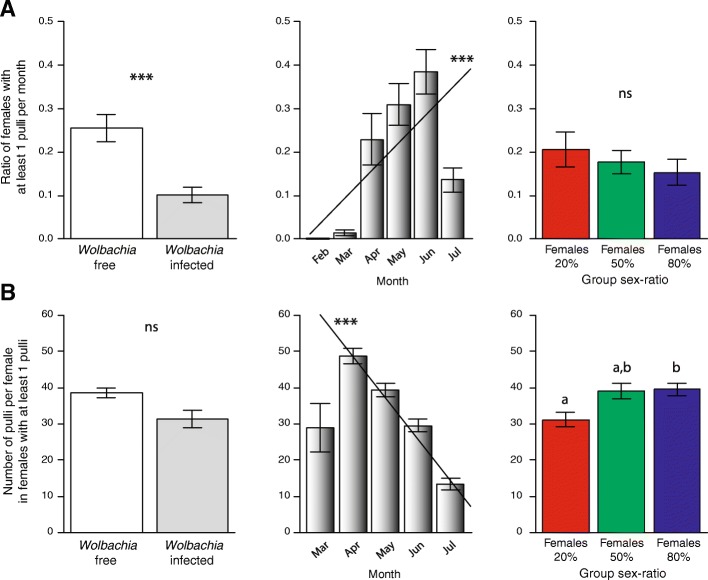
Proportion of females producing at least one descendant (**a**) and mean number of descendants in these females (**b**) as a function of *Wolbachia* infection (left), month of observation (middle) and group sex-ratio (right). Different letters correspond to *P* < 0.05; *** *P* < 0.0001

The proportion of females (alive) producing at least one pullus during our monthly observations was overall higher in *Wolbachia*-free compared to *Wolbachia*-infected females (Fig. 3a; LR χ^2^_1_ = 18.91, *P* < 0.0001), as well as overall increased over time (LR χ^2^_1_ = 44.22, *P* < 0.0001; model estimate ± SE = 1.00 ± 0.15). However, this proportion was independent of group sex-ratio (LR χ^2^_2_ = 1.94, *P* = 0.380) and of any interaction among the three tested factors (all *P* > 0.0709).

Overall, the mean number of descendants produced by each of these reproductive females (i.e., producing at least one pullus) decreased over the season (Fig. 3b; LR χ^2^_1_ = 82.80, *P* < 0.0001; model estimate ± SE = − 0.88 ± 0.09) and reflected group sex-ratio (LR χ^2^_2_ = 17.76, *P* = 0.0001). In particular, this mean number was higher in Females _80%_ compared to Females _20%_ (Tukey contrasts, *P* = 0.0001), whereas there was no difference between Females _20%_ and Females _50%_ (*P* = 0.1049) and between Females _50%_ and Females _80%_ (*P* = 0.0914). In contrast, the mean number of descendants produced by these reproductive females was independent of *Wolbachia* infection (Fig. 3b; LR χ^2^_1_ = 0.18, *P* = 0.6711). It is noteworthy that the triple interaction between *Wolbachia*, month and group sex ratio was not tested due to the lack of descendants produced by *Wolbachia*-infected females for the first 3 months.

## Discussion

To our knowledge, our experimental work is the first to explore the long term and (possibly) interactive effects of group sex-ratio and *Wolbachia*-infections on the survival, fertility and fecundity of females. First, our results reveal that *Wolbachia* infection shapes the effects of both population sex-ratio and reproductive season on female fecundity. Compared to *Wolbachia*-free females, *Wolbachia*-infected females exhibited a lower likelihood of being gravid when surrounded by a lack of males and did not exhibit the otherwise negative effect of seasons on this likelihood. Independent of *Wolbachia* infection, we then found that an increase in male density was overall costly for female fitness, as it decreased both their lifespan and their number of offspring. *Wolbachia*-infected females, on the other hand, suffered from the presence of a high proportion of males in the group, as it reduced the number of females that produced descendants. *Wolbachia* infection also reduced the likelihood of females producing at least 1 offspring, whereas it did not affect the number of pulli produced (when females had at least 1 descendant) and female lifespan. Finally, our results demonstrate that the proportion of females producing at least one descendant and the mean number of descendants for these females overall increased and decreased during the reproductive season, respectively.

We showed that infection by *Wolbachia* altered both the dynamics of female reproduction over 6 months and the effect of male sex-ratio on their fecundity. In *Wolbachia*-free females, the proportion of gravid females decreased over the reproductive season and was overall independent of male sex-ratio, whereas in *Wolbachia-*infected females, this proportion was constant during the reproductive season and overall lower in groups with a lack of males. This absence of seasonal-effect on the fecundity of *Wolbachia*-infected females likely reflects the major reduction in female fecundity observed at the beginning of the reproductive season, which remained constant and low over time. The second effect suggests that *Wolbachia*-infection made females more sensitive to the risk of remaining virgin in groups where they dramatically outnumbered males. This finding is supported by previous studies showing that sperm depletion occurs in *A. vulgare* males but only affects the fertility of *Wolbachia-*infected females [27], revealing that *Wolbachia-*infected females are less attractive than *Wolbachia-*free females and such pattern is correlated with different cuticular odor profiles [18] and demonstrating that males perform fewer interactions, fewer copulations and less sperm transfer to *Wolbachia-*infected compared to *Wolbachia-*free females [17]. Therefore, *Wolbachia* infection triggered the negative effects of group sex-ratio on female fecundity, but somewhat surprisingly, these effects did not accentuate the costs of sexual harassment for females due to male-biased sex-ratios but instead increased the risk of remaining virgin in groups exhibiting a female-biased sex-ratio.

Independent of *Wolbachia*-infection, our results reveal that females living with an excess of males exhibited a shorter lifespan between March and August (i.e., the onset of reproduction), as well as had lower overall reproductive success compared to the other females. Comparable effects of male-biased sex-ratios on female reproductive success have been reported in guppies and fruit flies, where females living in groups with a male-biased sex-ratio produced fewer offspring and suffered more from sexual harassment by males [28, 29]. Similarly, in guppies, the observed decrease in direct fitness was linked to sexual harassment of females and reflected their resulting reduced allocation of time to foraging behavior [30, 31]. Our results suggest that sexual harassment by males occurred in *A. vulgare* and shed light on the associated fitness costs for females as suggested in a previous study [25]. Interestingly, a few observations conducted during the experiment showed that two males tried to copulate simultaneously with one female in groups with high male density, whereas this was not observed in groups with low male density.

Notwithstanding the effects of group sex-ratio reported above, our results reveal that *Wolbachia*-infection reduced the likelihood of females producing at least one offspring and had no effect on their total number of descendants (when produced) and on female longevity. It is generally known that the effects of *Wolbachia* on host fitness greatly varies between host species, *Wolbachia* strains and measured fitness traits. For instance, *Wolbachia* decreased fertility but increased the life span of individuals if they were maintained on sugar meals in *Anopheles stephensi*, which is not a natural host of *Wolbachia* [32]. Conversely, in *Drosophila melanogaster*, some *Wolbachia* strains enhanced the costs of reproduction of females living with males [33]. Somewhat surprisingly, however, we showed that *Wolbachia* did not affect the longevity of its host. This result is in contrast with findings from a previous study, in which the authors monitored the survival of 100 *Wolbachia*-free females and 100 females infected with *Wolbachia,* and documented a lower survival for *Wolbachia-*infected females [16]. However, females in this previous study were isolated with one male for reproduction. Therefore, one likely explanation for the apparent differences between these two results is the effect of social isolation (*A. vulgare* is a gregarious species), which could have hindered their investment in immunity [34] and thus strengthened the negative effects of *Wolbachia* on female fitness. Future studies will investigate this hypothesis.

## Conclusions

To conclude, our study is the first to investigate the influence of group sex-ratio and a feminizing sex-ratio distorter on female survival and reproduction. Males, especially in a male biased sex-ratio group, appear to impact female survival and fertility, and a recent study showing that fewer females are found in natural populations at the end of the breeding season could indicate that these costs also exist under natural environments [35]. *Wolbachia* infection also has a strong negative impact on its host’s reproductive success. Overall, our findings highlight the importance of social pressures and infection on female fitness and provide insights into our general understanding of the joint and/or opposite effects of these two parameters on the evolution of reproductive strategies.

## Methods

### Animal rearing and experimental design

All *A. vulgare* individuals (Isopoda, Oniscidea, Latreille, 1804) used in this experiment originated from a *Wolbachia-*free population sampled in Denmark and then were maintained under a standard laboratory condition preventing sib-mating. The *Wolbachia-*infected females were descendants of mothers from this population, which were initially injected with the *Wolbachia w*VulC strain [36] and then naturally transmitted their bacteria to the next generations (hereafter, the *Wolbachia-*infected population). The descendants of these females are therefore feminized males and/or females [37]. In addition, all the males and *Wolbachia*-free females used in our experiments were derived from the *Wolbachia*-free population. All gravid females were isolated, their offspring then sexed, and the resulting males and females separated into different boxes before sexual maturity. All our experimental groups (see below) were set up with virgin adults that were 5–6 months old. All animals were reared in boxes (26 × 13 cm) on a substrate of moistened soil, provided with ad libitum food (dead leaves and slices of fresh carrots) and maintained at 20 °C under the natural photoperiod of Poitiers (France).

### Effects of group sex-ratio and *Wolbachia* infection on the survival rate and reproductive success of females

To test whether group sex-ratio and/or *Wolbachia* infection influence the survival and reproduction of females, we set up four types of groups with different sex-ratios: 100% female (15 females and 0 males), 80% female (15 females and 4 males), 50% female (15 females and 15 males) and 20% female (15 females and 60 males), hereafter called females _100%_, females _80%_, females _50%_, and females _20%_, respectively. For each sex-ratio, we set up five groups including *Wolbachia-*free females and five groups including *Wolbachia-*infected females. It is important to note that *A. vulgare* females can produce unfertilized eggs if they are not in contact with any males (Richard FJ, personal communication), which was therefore expected to occur in the females _100%_ treatment. To avoid covariation between animal density and experimental groups, the box dimensions were proportional to the number of animals inside, i.e., 17.5 × 11.5, 17.5 × 11.5, 35 × 23 and 70 × 46 cm, respectively. All experimental groups were then maintained under standard laboratory conditions (see above) for the following 12 months. To maintain stable sex-ratios over the experimental period, dead males were replaced by living males, whereas living males were removed from the groups where females died during these 12 months.

Female mortality was recorded on a monthly basis over the 12 months of the experiment, whereas changes in the number of gravid females per group and the total number of juveniles (called pulli) produced per female were measured over the first 6 months of the experiment, which corresponds to the reproductive period of this species. To follow the number of pulli produced per female over the reproductive season, one subset of gravid females was isolated each month in a small individual box until the birth of their pulli (i.e., just a few days). These pulli were counted, and their mothers were placed back into their experimental group. Each of these gravid females was marked on its cuticle with a Posca® marker before its isolation to avoid their reuse in subsequent measurements of their groups.

### Data analyses

All statistical analyses were conducted using R v3.4.3 (https://cran.r-project.org/) with the *survival*, *lme4, emmeans* and *car* packages. Initially, the survival rate of females was analyzed at 6 months (i.e., over the reproductive period) and at 12 months (i.e., over the entire experiment), that is, females still alive after six or 12 months, respectively, after the beginning of the experiment using two Cox proportional hazard regression models allowing for censored data. In these models, group sex-ratio (females _100%_, females _80%,_ females _50%_, or females _20%_) and *Wolbachia* infection (*Wolbachia*-free or *Wolbachia*-infected) were the explanatory variables. To control for the nonindependence of females maintained in the same rearing boxes, we also entered the box-ID into each statistical model using the *frailty* argument.

We analyzed the proportion of gravid females (females’ fecundity) using a Generalized Linear Mixed Model (GLMER) with binomial error distribution and correction for overdispersion. In this model, the proportion of gravid females was entered as a response variable (using the *cbind* function in R), whereas group sex-ratio, *Wolbachia* infection and the month of observation (as a continuous value) were used as explanatory variables. The use of month as a continuous value allowed us testing whether *Wolbachia*-infection alters the negative effect of time on the proportion of gravid female per months. Because several females were maintained in the same rearing boxes, we also used the box-ID as a random effect.

Many females did not produce any pulli during the experiment, so female fertility was investigated in two ways. In the first way, we tested whether the proportion of females with at least 1 pullus per group reflected group sex-ratio, *Wolbachia* infection and the month of observation. To this end, we used a GLMER with binomial error distribution and correction for data overdispersion and entered the box-ID as a random effect. In the second step, we tested whether the number of pulli produced by these females (i.e., females with at least 1 pullus) reflected group sex-ratio, *Wolbachia* infection and the month of observation. Here, we used a general Linear Mixed Model (LMER) and entered the box-ID as a random effect. It is important to note that these two analyses did not include Females _100%_, as all these females were virgin and were consequently unable to produce fertilized eggs.

All statistical models initially included all possible interactions between the tested variables and were then simplified via the stepwise deletion of nonsignificant interactions (all *P* < 0.05). Pairwise comparisons between group sex-ratio were tested using model contrasts and *P*-values were corrected for multiple testing using Tukey corrections.

## Additional files

Additional file 1: **Table S1.** Full model. The proportion of gravid females was shaped by a triple interaction between *Wolbachia* infection, group sex-ratio and month. The full model results of the measured parameters are presented. (DOCX 12 kb)

Additional file 2: S2 Raw data. Data availability The excel file provides all the raw data collected in the current study. (XLSX 48 kb)

## Abbreviations

GLMER: Generalized Linear Mixed Model
LMER: General Linear Mixed Model
LR: Likelihood Ratio
