# Supplementary material for: Joint effects of group sex-ratio and Wolbachia infection on female reproductive success in the terrestrial isopod Armadillidium vulgare
Source: BMC Evol Biol. 2019 Feb 28;19:65. doi: 10.1186/s12862-019-1391-6 (PMC6394025; doi:10.1186/s12862-019-1391-6)
Supplement: Supplementary file 1 — Table S1. Full model. The proportion of gravid females was shaped by a triple interaction between Wolbachia infection, group sex-ratio and month. The full model results of the measured parameters are presented. (DOCX 12 kb) [file 12862_2019_1391_MOESM1_ESM.docx]

SUPPLEMENTARY MATERIAL

**Table S1** –A triple interaction between sex-ratio, *Wolbachia* infection and month shaped the proportion of gravid females. Significant p-values are in bold.

|  | LR χ^2^ | Df | P |
| --- | --- | --- | --- |
| Sex-ratio (SR) | 4.20 | 3 | 0.2411 |
| Wolbachia | 22.35 | 1 | **<0.0001** |
| Month | 1.62 | 1 | 0.2031 |
| SR:Wolbachia | 7.72 | 3 | 0.0521 |
| SR:Month | 1.86 | 3 | 0.6021 |
| Wolbachia:Month | 10.62 | 1 | **0.0011** |
| SR:Wolbachia:Month | 8.96 | 3 | **0.0298** |
